# Tumour suppressive *microRNA-874* regulates novel cancer networks in maxillary sinus squamous cell carcinoma

**DOI:** 10.1038/bjc.2011.311

**Published:** 2011-08-16

**Authors:** N Nohata, T Hanazawa, N Kikkawa, D Sakurai, L Fujimura, T Chiyomaru, K Kawakami, H Yoshino, H Enokida, M Nakagawa, A Katayama, Y Harabuchi, Y Okamoto, N Seki

**Affiliations:** 1 Department of Functional Genomics, Chiba University Graduate School of Medicine, 1-8-1 Inohana Chuo-ku, Chiba 260-8670, Japan; 2 Department of Otorhinolaryngology/Head and Neck Surgery, Chiba University Graduate School of Medicine, Chiba, Japan; 3 Biomedical Research Center, Chiba University, Chiba, Japan; 4 Department of Urology, Graduate School of Medical and Dental Sciences, Kagoshima University, Kagoshima, Japan; 5 Department of Otorhinolaryngology/Head and Neck Surgery, Asahikawa Medical University, Asahikawa, Japan

**Keywords:** microRNA, *miR-874*, tumour suppressor, *PPP1CA*, maxillary sinus

## Abstract

**Background:**

On the basis of the microRNA (miRNA) expression signature of maxillary sinus squamous cell carcinoma (MSSCC), we found that *miR-874* was significantly reduced in cancer cells. We focused on the functional significance of *miR-874* in cancer cells and identification of *miR-874*-regulated novel cancer networks in MSSCC.

**Methods:**

We used PCR-based methods to investigate the downregulated miRNAs in clinical specimens of MSSCC. Our signature analyses identified 23 miRNAs that were significantly reduced in cancer cells, such as *miR-874*, *miR-133a*, *miR-375*, *miR-204*, and *miR-1*. We focused on *miR-874* as the most downregulated novel miRNA in our analysis.

**Results:**

We found potential tumour suppressive functions such as inhibition of cancer cell proliferation and invasion. A molecular target search of *miR-874* revealed that *PPP1CA* was directly regulated by *miR-874*. Overexpression of *PPP1CA* was observed in MSSCC clinical specimens. Silencing of the *PPP1CA* gene significantly inhibited cancer cell proliferation and invasion.

**Conclusion:**

The downregulation of *miR-874* was a frequent event in MSSCC, which suggests that *miR-874* functions as a tumour suppressive miRNA, directly regulating *PPP1CA* that has a potential role of an oncogene. The identification of novel *miR-874*-regulated cancer pathways could provide new insights into potential molecular mechanisms of MSSCC oncogenesis.

The maxillary sinuses are single pyramidal cavities in the body of the maxillae. Squamous cell carcinoma is the most common cancer of the maxillary sinus (60–70%), followed by adenoid cystic carcinoma (Million and Cassisi, 1994). Maxillary sinus squamous cell carcinoma (MSSCC) comprises 2–3% of all head and neck tumours and the annual incidence is 0.5–1.0 per 100 000 people (Tiwari *et al*, 2000; Dulguerov and Allal, 2006). Clinical symptoms of MSSCC present insidiously, and sometimes resemble those of chronic sinusitis. Although presentation of lymph node and distant metastasis were uncommon, primary tumours are often diagnosed as advanced disease. The 5-year survival rate of T4 tumours is ∼50%. Local recurrence is the most common cause of treatment failure and death (Konno *et al*, 1998; Tiwari *et al*, 2000; Dulguerov and Allal, 2006).

From an epidemiological standpoint, occupational exposures to leather, wood dust, nickel, arsenic, and formaldehyde have been implicated in the aetiology of MSSCC (Luce *et al*, 2002; Bornholdt *et al*, 2008). In contrast, tobacco, a major risk factor for head and neck squamous cell carcinoma (HNSCC) does not have an important role in MSSCC (Zheng *et al*, 1993; Holt, 1994). It has been suggested that molecular mechanisms of carcinogenesis might be different for these cancers (Lopez *et al*, 2011). Although analyses of major cancer-related genes, such as TP53 and K-ras, mutation were reported (Bornholdt *et al*, 2008; Holmila *et al*, 2010), relatively few genome-wide gene expression analyses of MSSCC have been conducted and no analyses of microRNAs (miRNAs) have been performed for this disease.

MicroRNAs are small non-coding RNAs of 20–22 nucleotides, and are involved in crucial biological processes, including development, differentiation, apoptosis, and proliferation through imperfect pairing with target mRNAs of protein-coding genes and the transcriptional or post-transcriptional regulation of their expression (Bartel, 2004). Bioinformatic predictions indicate that miRNAs regulate >30% of the protein coding genes (Filipowicz *et al*, 2008). Currently, 1424 human miRNAs are registered at miRBase release 17.0 (http://microrna.sanger.ac.uk/).

Recent studies suggest that miRNAs contribute to the initiation and development of various types of cancer (Calin and Croce, 2006). Some highly expressed miRNAs could function as oncogenes by repressing tumour suppressor genes, whereas low-expressed miRNAs could function as tumour suppressors by negatively regulating oncogenes (Esquela-Kerscher and Slack, 2006). We have conducted searches of tumour suppressive miRNAs based on analyses of expression signatures of various cancers (Ichimi *et al*, 2009; Kano *et al*, 2010; Kikkawa *et al*, 2010; Yoshino *et al*, 2011). These studies successfully identified several tumour suppressive miRNAs such as *miR-1*, *miR-133a*, and *miR-145* (Chiyomaru *et al*, 2010b; Kano *et al*, 2010; Yoshino *et al*, 2011; Nohata *et al*, 2011b). The next major problem is the identification of the oncogenes that are controlled by these miRNAs. Our recent studies showed that several target genes did indeed function as oncogenes (Chiyomaru *et al*, 2010a, 2011; Fuse *et al*, 2011; Mutallip *et al*, 2011; Nohata *et al*, 2011a).

The aim of this study was to identify new tumour suppressive miRNAs revealed in our MSSCC expression analysis. We focused on *miR-874*, which was the most significantly downregulated miRNA in our signature. We found that it functioned as a tumour suppressor based on our findings of inhibited cancer cell proliferation and invasion. Genome-wide expression analysis identified several candidate target genes such as *PPP1CA* (protein phosphatase 1, catalytic subunit, a isozyme), *PAAF1* (proteasomal ATPase-associated factor 1), and *TGOLN2* (trans-Golgi network (TGN) protein 2). Insights into the association between tumour suppressive *miR-874* and their target oncogene networks could enhance our understanding of the molecular mechanism of MSSCC carcinogenesis.

## Materials and methods

### Clinical MSSCC specimens

In all, 20 pairs of primary MSSCC and corresponding normal epithelial samples were obtained from patients with MSSCC in Chiba University Hospital (Chiba, Japan) from 2005 to 2010. The fresh specimens were immediately immersed in RNAlater (Qiagen, Valencia, CA, USA) and stored at −20 °C until RNA was extracted. The samples macroscopically considered normal were confirmed free of cancer cells by microscopic pathological examination. The patients' backgrounds and clinicopathological characteristics are summarised in Table 1. The patients were classified according to 2002 Union for International Cancer Control TNM staging criteria (Sobin and Wittekind, 2002) before treatment. Written consent of tissue donation for research purposes was obtained from each patient before tissue collection. The protocol was approved by the Institutional Review Board of Chiba University.

### RNA isolation

Total RNA was isolated using TRIzol reagent (Invitrogen, Carlsbad, CA, USA) according to the manufacturer's protocol. RNA concentrations were determined spectrophotometrically, and molecular integrity was checked by gel electrophoresis. RNA quality was confirmed using an Agilent 2100 Bioanalyzer (Agilent Technologies, Santa Clara, CA, USA).

### MicroRNA expression signatures and data normalisation

Tissue specimens for miRNA screening using a low density array (LDA) were from five MSSCC patients at Chiba University Hospital between 2005 and 2007 (Table 1; #1–#5). The miRNA expression patterns were evaluated using the TaqMan LDA Human microRNA Panel v2.0 (Applied Biosystems, Foster City, CA, USA). The assay was composed of two steps: generation of cDNA by reverse transcription (RT) and a TaqMan real-time PCR assay. Description of real-time PCR and the list of human miRNAs can be found on the company's website (http://www.appliedbiosystems.com). Analysis of relative miRNA expression data was performed using GeneSpring GX version 7.3.1 software (Agilent Technologies) according to the manufacturer's instructions. A cutoff *P*-value of <0.05 was used to narrow down the candidates after global normalisation of the raw data. After global normalisation, additional normalisation was carried out with *RNU48*.

### Maxillary sinus squamous cell carcinoma cell culture

The human MSSCC cell line IMC-3 (Mizoguchi *et al*, 1991) was used. Cells were grown in RPMI 1640 medium supplemented with 10% fetal bovine serum in a humidified atmosphere containing 5% CO_2_ at 37 °C.

### Mature miRNA transfection and small-interfering RNA treatment

The following RNA species were used in this study: mature miRNAs, pre-miR miRNA precursors (hsa-*miR*-*874*; pre-miR ID: PM12355), negative control miRNA (P/N: AM17111) (Applied Biosystems), small-interfering RNA (Stealth Select RNAi siRNA; si-*PPP1CA*_1 Cat# HSS143413, si-*PPP1CA*_2 Cat# HSS143414) (Invitrogen), and negative control siRNA (Stealth RNAi Negative Control Medium GC Duplex; 12935–300) (Invitrogen). RNAs were incubated with Opti-MEM (Invitrogen) and Lipofectamine RNAiMax reagent (Invitrogen) as described previously (Ichimi *et al*, 2009). Transfection efficiency of pre-miR in cell lines was confirmed based on downregulation of *TWF1* (*PTK9*) mRNA following transfection with *miR-1* as previously reported (Ichimi *et al*, 2009).

### Cell proliferation assays

Cells were transfected with 10 nM miRNA and siRNA by RT and plated in 96-well plates at 3 × 10^3^ cells per well. After 72 h, cell proliferation was determined by the XTT assay, using the Cell Proliferation Kit II (Roche Molecular Biochemicals, Mannheim, Germany) as previously reported (Ichimi *et al*, 2009; Chiyomaru *et al*, 2010b). Triplicate wells were measured for cell viability in each treatment group. Furthermore, we also conducted counting number of cells with each treatment. Cells (1 × 10^4^) were incubated in a 24-well plate for 24, 48, and 72 h. They were then treated with trypsin and stained with trypan blue. Viable cells, which excluded trypan blue dye, were counted in quadruplicate with a Countess (Invitrogen).

### Cell invasion assays

A cell invasion assay was carried out using modified Boyden chambers containing transwell-precoated Matrigel membrane filter inserts with 8 *μ*m pores in 24-well tissue culture plates at 1 × 10^5^ cells per well (BD Biosciences, Bedford, MA, USA; Kano *et al*, 2010; Chiyomaru *et al*, 2010b). Triplicate wells were measured for cell invasion in each treatment group.

### Target gene search for *miR-874*

Genome-wide screens using *miR-874* transfectants were performed to identify target genes of *miR-874* in IMC-3. Oligo-microarray human 44K (Agilent Technologies) was used for expression profiling of the transfectants in comparison with a miRNA-negative control transfectant. Hybridisation and wash steps were performed as previously described (Sugimoto *et al*, 2009). The arrays were scanned using a Packard GSI Lumonics Scan Array 4000 (Perkin Elmer, Boston, MA, USA). The data were analysed by means of DNASIS array software (Hitachi Software Engineering, Tokyo, Japan), which converted the signal intensity for each spot into text format. The log_2_ ratios of the median subtracted background intensities were analysed. Data from each microarray study were normalised by a global normalisation method. Predicted target genes and their target miRNA binding site seed regions were investigated using TargetScan (release 5.1, http://www.targetscan.org/). The sequences of the predicted mature miRNAs were confirmed using miRBase (release 17.0, http://microrna.sanger.ac.uk/).

### Quantitative real-time RT–PCR

First-strand cDNA was synthesised from 1 *μ*g of total RNA using a High Capacity cDNA Reverse Transcription Kit (Applied Biosystems). Gene-specific PCR products were assayed continuously using a 7900-HT Real-Time PCR System according to the manufacturer's protocol. The initial PCR step consisted of a 10 min hold at 95 °C, followed by 40 cycles consisting of a 15 s denaturation at 95 °C and a 1 min annealing/extension at 63 °C. TaqMan probes and primers for *PPP1CA* (P/N: Hs00267568_m1), *PAAF1* (P/N: Hs00228523_m1), *TGOLN2* (P/N: Hs00197728_m1) and *GUSB* (P/N: Hs99999908_m1) internal control were obtained from Applied Biosystems (Assay-On-Demand Gene Expression Products). The expression levels of *miR-874* (assay ID: 002268) were analysed by TaqMan quantitative real-time PCR (TaqMan MicroRNA Assay; Applied Biosystems) and normalised to *RNU48* (assay ID: 001006). The ΔΔ*C*_t_ method was adopted and applied to calculate relative quantity of subject genes. All reactions were performed in triplicate, and included negative control reactions that lacked cDNA.

### Western blots

Cells were harvested at 72 h after transfection and lysates were prepared. A 50 *μ*g of protein lysate was separated by NuPAGE on 4–12% bis-tris gels (Invitrogen) and transferred to PVDF membranes. Immunoblotting was performed with diluted (1:200) monoclonal protein phosphatase 1 *α* (PP1*α*) antibody (sc-7482; Santa Cruz Biotechnology, Santa Cruz, CA, USA), with *β*-actin antibody (sc-1615; Santa Cruz Biotechnology) used as an internal control. The membrane was washed and incubated with goat anti-mouse IgG (H+L)–HRP conjugate (Bio-Rad, Hercules, CA, USA). Specific complexes were visualised by echochemiluminescence (GE Healthcare Bio-Sciences, Princeton, NJ, USA), and the expression levels of these genes were evaluated by ImageJ software (version 1.44; http://rsbweb.nih.gov/ij/).

### Plasmid construction and dual-luciferase reporter assay

The wild-type sequences of *PPP1CA* 3′-UTR and those with deleted *miR-874* target sites (position 237–243) were inserted between the *Xho*I–*Pme*I restriction sites in the 3-UTR of the *hRluc* gene in psiCHECK-2 vector (C8021; Promega, Madison, WI, USA). Sequences of oligonucleotides are described in the Supplementary Information. The synthesised DNA was cloned into the psiCHECK-2 vector. The IMC-3 cells were transfected with 15 ng of vector, 10 nM of *miR-874* (Applied Biosystems), and 1 *μ*l of Lipofectamine 2000 (Invitrogen) in 100 *μ*l of Opti-MEM (Invitrogen). The activities of firefly and *Renilla* luciferases in cell lysates were determined with a dual-luciferase assay system (E1910; Promega). Normalised data were calculated as the quotient of *Renilla*/firefly luciferase activities.

### Statistical analysis

The relationships between two groups and the numerical values obtained by real-time RT–PCR were analysed using the non-parametric Mann–Whitney *U*-test or the paired *t*-test. The relationship among three variables and numerical values was analysed using the Bonferroni adjusted Mann–Whitney *U*-test. Spearman's rank test was used to evaluate the relationships among the relative expression levels of *miR-874*, *PPP1CA*, *PAAF1*, and *TGOLN2* mRNA. All analyses were performed using Expert StatView (version 4, SAS Institute Inc., Cary, NC, USA).

## Results

### Identification of downregulated miRNAs in MSSCC by miRNA expression signature: expression of *miR-874* in MSSCC clinical specimens

**Figure 1:**
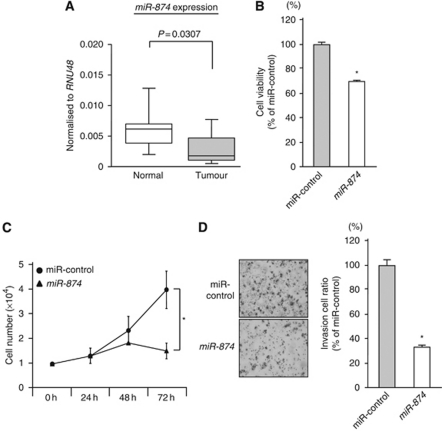
Expression of *miR-874* in MSSCC clinical specimens and gain-of-function study using *miR-874* in the IMC-3 cell line. (**A**) The *miR-874* expression levels in clinical specimens. Real-time RT–PCR showed that miRNA expression in tumour tissues was lower than that of normal tissues. *RNU48* was used as an internal control. (**B**) Cell proliferation determined by the XTT assay in the IMC-3 cell line transfected with 10 nM of *miR-874* or miR-control. (**C**) Cell number was counted after transfection with 10 nM of miR-874 or miR-control at 24, 48, and 72 h. (**D**) Cell invasion activity determined by the Matrigel invasion assay in IMC-3 cell lines transfected with 10 nM of *miR-874* or miR-control. ^*^P<0.05.

We evaluated mature miRNA expression levels of five pairs of normal epithelia and MSSCC by miRNA expression signature analysis. In all, 23 significantly downregulated miRNAs were selected after normalisation to *RNU48* (Table 2). The *miR-874*, the most downregulated miRNA in the list, was selected for further study. Quantitative stem–loop RT–PCR demonstrated that the expression levels of *miR-874* were significantly lower in 20 MSSCC specimens in comparison with normal tissues (*P*=0.0307, Figure 1A).

### Effect of *miR-874* transfection on the proliferation and invasion of IMC-3

To investigate the functional roles of miR-874, we performed gain-of-function studies using miRNA transfection of IMC-3.

The XTT assay showed significant inhibition of cell proliferation in *miR-874* transfectants in comparison with the miR-control transfectants (% of cell proliferation, 69.6±0.8 and 100.0±3.3, respectively; *P*<0.05; Figure 1B). This result was also confirmed by performing cell counting assay (cell number, 1.5 × 10^4^±2.9 × 10^3^ and 3.7 × 10^4^±6.9 × 10^3^, respectively; *P*<0.05; Figure 1C).

The Matrigel invasion assay demonstrated that invading cell numbers were significantly decreased in miR-874 transfected IMC-3 cells in comparison with the controls (% of cell invasion, 31.3±6.9 and 100.0±11.2, respectively, *P*<0.05; Figure 1C).

### Gene expression profile identifies downregulated genes in *miR-874* transfectants

To gain further insight into which genes were affected by *miR-874* transfection, we performed gene expression analysis with *miR-874* transfectants and the controls in IMC-3 cells. Signal values of raw data in miR-control transfectants <5000 were cutoff. A total of 18 genes were downregulated less than −1.0 (log_2_ ratio) in *miR-874* transfectants compared with the controls. The TargetScan programme showed that seven of the genes had putative target sites of *miR-874* in their 3′-UTR (Table 3). Entries from the microarray data were approved by the Gene Expression Omnibus (GEO) and were assigned GEO accession number GSE19714.

### Expression levels of candidate target genes of *miR-874* in MSSCC clinical specimens

**Figure 2:**
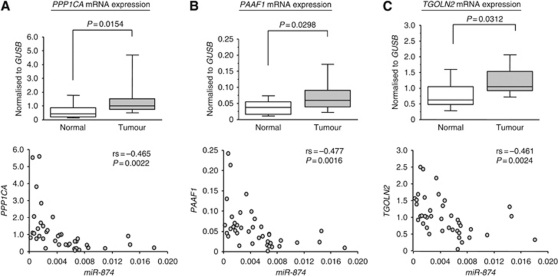
Expression levels of three candidate genes of *miR-874* target were measured by real-time RT–PCR. (**A**, upper) *PPP1CA* mRNA expression levels in MSSCC clinical specimens. (**B**, upper) *PAAF1* mRNA expression levels in MSSCC clinical specimens. (**C**, upper) *TGOLN2* mRNA expression levels in MSSCC clinical specimens. Real-time RT–PCR showed that each of the three genes in tumour tissues was expressed at higher levels than that in the normal tissues. *GUSB* was used as an internal control. (**A**, **B**, and **C**, lower) Significant inverse correlations between each of the genes and the level of *miR-874* expression were shown.

We measured the mRNA expression levels of seven candidate genes in MSSCC clinical specimens by quantitative real-time RT–PCR. Three genes, *PPP1CA*, *PAAF1*, and *TGOLN2* were significantly upregulated in cancer tissues (*P*=0.0154, *P*=0.0298, and *P*=0.0312 respectively; Figure 2A, B and C, upper panel). The other four genes (*CPS1*, *COL12A1*, *EIF3H*, and *NBR1*) were not upregulated in the tumour region of MSSCC (Supplementary Figure). There were significant inverse correlations between each of the genes and the level of *miR-874* expression (Figure 2A, B and C, lower panel).

### *PPP1CA* is directly regulated by *miR-874*

**Figure 3:**
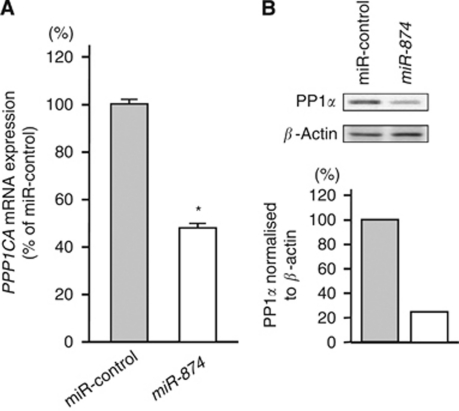
*PPP1CA* mRNA and PP1*α* protein expression in the IMC-3 cell line. (**A**) *PPP1CA* mRNA expression 48 h after transfection with 10 nM with *miR-874*. *PPP1CA* mRNA expression was significantly repressed in *miR-874* transfectants. *GUSB* was used as an internal control. (**B**) PP1*α* protein expression 72 h after transfection with *miR-874*. *β*-Actin was used as a loading control. The protein expression level of PP1*α* was also repressed in *miR-874* transfectants.

**Figure 4:**
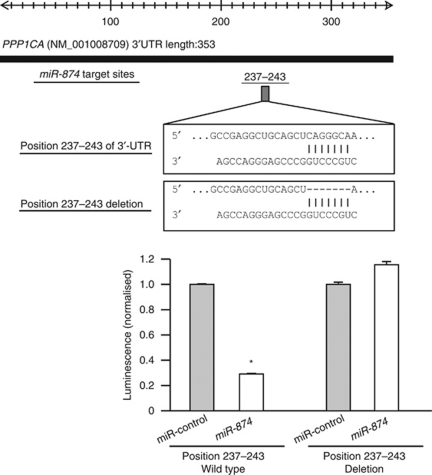
Schematic of conserved binding sites for *miR-874* (upper). Putative conserved target site in the *PPP1CA* 3′-UTR was identified with the TargetScan database: the one *miR-874* target site is indicated. IMC-3 cells were transfected with 15 ng of *PPP1CA* 3′-UTR in a vector construct and 10 nM of *miR-874* or miR-control. Vectors were used with encoding the entire sequence of 3′-UTR of *PPP1CA* mRNA or that with a deletion of the *miR-874* target (position 237–243). Renilla luciferase activity was measured after a 24 h transfection. The results were normalised against firefly luciferase values (lower). ^*^P<0.05.

*PPP1CA* mRNA and PP1*α* protein expression levels were markedly downregulated in the *miR-874* transfectants in comparison with the controls (Figure 3A and B). We performed a luciferase reporter assay to determine whether *PPP1CA* mRNA had a target site for *miR-874*. We used a vector encoding either the total sequence of the 3′-UTR of *PPP1CA* mRNA, including the predicted *miR-874* target site (positions 237–243), or a vector lacking the *miR-874* target site. We found that the luminescence intensity was significantly reduced by transfection of the entire 3′-UTR of *PPP1CA*, whereas deletion of positions 237–243 blocked the decrease in luminescence (Figure 4).

### Effect of *PPP1CA* silencing on cell proliferation and invasion in IMC-3

**Figure 5:**
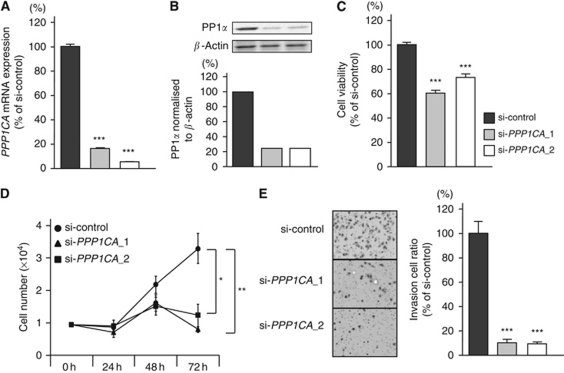
Effect of si-*PPP1CA* in the IMC-3 cell line. (**A**) *PPP1CA* mRNA expression at 48 h after transfection with 10 nM of si-*PPP1CA*_1, si-*PPP1CA*_2, or si-control. *PPP1CA* mRNA expression was repressed in si-*PPP1CA*_1 and si-*PPP1CA*_2 transfectants. *GUSB* was used as an internal control. (**B**) PP1*α* protein expression at 72 h after transfection of the siRNAs. *β*-Actin was used a loading control. The protein expression level of PP1*α* was also repressed in si-*PPP1CA*_1 and si-*PPP1CA*_2 transfectants. (**C**) Cell proliferation determined with the XTT assay in the IMC-3 cell line transfected with 10 nM of si-*PPP1CA*_1, si-*PPP1CA*_2, or si-control. (**D**) Cell number was counted after transfection with 10 nM of si-*PPP1CA*_1, si-*PPP1CA*_2, or si-control at 24, 48, and 72 h. (**E**) Cell invasion activity determined with the Matrigel invasion assay in the IMC-3 cell line transfected with 10 nM of si-*PPP1CA*_1, si-*PPP1CA*_2, or si-control. ^*^P<0.0018, ^**^P<0.0005, and ^***^P<0.0001.

To examine the functional role of *PPP1CA*, we performed loss-of-function studies using two different si-*PPP1CAs* transfected into the IMC-3 cell line. The *PPP1CA* mRNA and PP1*α* protein expression levels were markedly reduced by the two different si-*PPP1CA* transfectants (Figure 5A and B).

The XTT assay revealed significant inhibition of cell proliferation in the two different si-*PPP1CA* transfectants in comparison with growth of the si-control transfectants (% of cell proliferation: 60.4±1.2, 73.2±1.0, and 100.0±5.0, respectively; *P*<0.0001; Figure 5C). This result was also confirmed by performing cell counting assay (cell number, 8.0 × 10^3^±1.2 × 10^3^, 1.4 × 10^4^±4.6 × 10^3^, and 3.2 × 10^4^±6.0 × 10^3^, respectively; *P*=0.0005 and *P*=0.0018, respectively; Figure 5D).

The Matrigel invasion assay demonstrated that the number of invading cells was significantly decreased in the two different si-*PPP1CA* transfectants compared with their counterparts (% of cell invasion, 11.5±2.8, 8.1±1.4, and 100.0±11.7, respectively; *P*<0.0001; Figure 5E).

## Discussion

This is the first article to investigate aberrant miRNA expression in MSSCC clinical specimens. The *miR-133a* and *miR-1* were among the top five downregulated miRNAs in our expression analysis. Interestingly, *miR-1-1*/*miR-133a-2*, and *miR-1-2*/*miR-133a-1* are clustered on different chromosomal regions in the human genome, 20q13.33 and 18q11.2, respectively. Recently, our analyses of oesophageal cancer and bladder cancer expression signatures confirmed downregulation of both miRNAs (Kano *et al*, 2010; Chiyomaru *et al*, 2010b), and we demonstrated that *miR-1* and *miR-133a* function as tumour suppressors in many types of cancers regulating several oncogenes (Chiyomaru *et al*, 2010a, 2010b; Kano *et al*, 2010; Mutallip *et al*, 2011; Nohata *et al*, 2011a, 2011b; Uchida *et al*, 2011; Yoshino *et al*, 2011). When we consider other miRNAs in this signature, *miR-145* downregulation has frequently been reported in cancers, including prostate, bladder, colon, ovarian, and oesophageal cancers as well as B-cell malignancies (Akao *et al*, 2007; Iorio *et al*, 2007; Arndt *et al*, 2009; Kano *et al*, 2010; Chiyomaru *et al*, 2010b; Zaman *et al*, 2010). The *miR-145* is located on chromosome 5q32–33 within a 4.09 kb region (http://microrna.sanger.ac.uk/). Of interest, 5q31.1 is a well-known fragile site in the human genome (http://www.genenames.org/) and is often deleted in cancers. Increasing evidence and our data indicate that *miR-145* functions as a tumour suppressive miRNA and inhibits cell growth, invasion, and migration in cancer cells (Kano *et al*, 2010; Chiyomaru *et al*, 2010b; Fuse *et al*, 2011). Our present analysis generated a list of sequences that could be involved in the pathology of MSSCC. Analysis of miRNAs included in this signature could enhance our understanding of MSSCC carcinogenesis.

In this study, we focused on the functional significance of *miR-874*, because it was the most downregulated miRNA in our signature and functional analysis of *miR-874* had not yet been reported. The *miR-874* was recently identified based on small RNA library sequencing and is conserved across most, but not all mammals (Landgraf *et al*, 2007; Lui *et al*, 2007). Our results showed that *miR-874* was downregulated in MSSCC cells and ectopic expression of *miR-874* significantly inhibited cell proliferation and invasion in IMC-3 cells. These results indicated that *miR-874* might function as a tumour suppressor in IMC-3 cells. Further studies are required to elucidate the precise mechanisms of *miR-874* regulation for initiation and development of MSSCC oncogenesis.

We performed a genome-wide analysis using *miR-874* transfected IMC-3 cells to elucidate the target genes regulated by *miR-874*. From the microarray analysis, we identified seven candidate genes (*CPS1*, *PPP1CA*, *PAAF1*, *COL12A1*, *EIF3H*, *TGOLN2*, and *NBR1*) containing *miR-874* target sites. To validate the mRNA expression levels of seven candidate genes in MSSCC clinical specimens by quantitative real-time RT–PCR, we narrowed down to three genes (*PPP1CA*, *PAAF1*, and *TGOLN2*) whose expression levels were significantly upregulated in MSSCC clinical specimens compared with normal tissues.

*PAAF1* inhibits proteasome 26S assembly and proteolytic activity by impairing the association of the 19S regulatory complex with the 20S core. The 26S proteasome consists of a 20S proteolytic core particle and 19S regulatory complexes. The 26S proteasome has an important role in ubiquitin-dependent proteolysis, which regulates many biological processes, such as cell cycle progression and signal transduction (Park *et al*, 2005). *TGOLN2*, *TGN* protein 2, is a cargo protein of retrograde transport, in which proteins and lipids are moved between endosomes and the TGN. Although the functions of several cargo proteins have been elucidated in retrograde transport, the role of TGOLN2 remains unknown (Johannes and Popoff, 2008; Pfeffer, 2009). These two genes currently have little association with cancer development. Therefore, we focused on *PPP1CA* as a subject of further experiment.

*PPP1CA* encodes the catalytic subunit of PP1*α*. The PP1*α* catalytic subunit can form complexes with many regulatory subunits, which regulate various cellular activities such as the cell cycle, apoptosis, and signal transduction (Cohen, 2002; Ceulemans and Bollen, 2004). Previous analysis of the protein showed that PP1*α* dephosphorylates the BRCA1 protein, coded by the tumour suppressor *BRCA1*, in breast and ovarian cancer. Those findings indicate that PP1*α* may have an oncogenic role (Liu *et al*, 2002). In addition, it has been reported that overexpression of PP1*α* is observed in pre-malignant hepatic cells and oral squamous cell carcinoma (Saadat *et al*, 1995; Imai *et al*, 1999; Hsu *et al*, 2006). In contrast, PP1*α* may function as a tumour suppressor by activating tumour suppressor protein pRB (Alberts *et al*, 1993). Our present data suggest that the *PPP1CA* gene functions as an oncogene in MSSCC. A molecular network search for downstream targets of *PPP1CA* in MSSCC will be necessary. Unfortunately, there was no significant relationship between *miR-874* or *PPP1CA* expression and clinicopathological parameters in this study. Our cohort was too small to evaluate this relationship. In addition, our samples are mostly at the late stage. Hence, a large-scale clinical test including the early stage samples will be necessary.

It is also of interest that *PPP1CA* is located at chromosomal region 11q13. Amplification of the chromosomal region on 11q13 is frequently observed in human cancers including HNSCC and breast cancer, and it is well known that *CCND1*, encoding cyclin D1, is a putative oncogene in the 11q13 amplicon (Schuuring, 1995; Schwab, 1998; Gollin, 2001). We recently demonstrated that when chromosomal region 11q13 was gained, the expression levels of several genes were elevated (Sugimoto *et al*, 2009). The oncogenic function of *PPP1CA* and amplification of this region may be related closely, so it will be important to examine the structural changes in this region in MSSCC.

In conclusion, the reduction of *miR-874* and increase of *PPP1CA* were frequent events in MSSCC cancer cells. The *miR-874* may function as a tumour suppressor and may directly regulate *PPP1CA*. The *miR-874* regulates novel cancer pathways and could provide new insights into molecular mechanisms in MSSCC and might contribute to the development of new therapeutic strategies for the disease.

## Figures and Tables

**Table 1:** Clinicopathological characteristics of 20 patients with maxillary sinus squamous cell carcinoma

**No.**	**Age (years)**	**Gender**	**Differentiation**	**T**	**N**	**M**	**Stage**
1	65	Female	Poor	4b	0	0	IVB
2	65	Male	Moderate	4a	0	0	IVA
3	74	Male	Well	4a	0	0	IVA
4	71	Male	Moderate	3	1	0	III
5	67	Male	Moderate	4a	0	0	IVA
6	68	Male	Well	4b	0	0	IVB
7	77	Male	Poor	3	0	0	III
8	76	Male	Moderate	3	0	0	III
9	61	Male	Well	3	0	0	III
10	54	Male	Poor	3	0	0	III
11	64	Male	Poor	4b	0	0	IVB
12	64	Male	Moderate	4a	0	0	IVA
13	80	Male	Moderate	4a	0	0	IVA
14	66	Female	Poor	4a	2c	0	IVA
15	60	Male	Poor	4a	0	0	IVA
16	66	Female	Moderate	4a	0	0	IVA
17	85	Male	Poor	4a	0	0	IVA
18	69	Male	Well	4a	0	0	IVA
19	57	Male	Poor	4a	0	0	IVA
20	69	Male	Poor	4a	2b	0	IVA

**Table 2:** Downregulated microRNAs in maxillary sinus squamous cell carcinoma (normalised to RNU48)

			**Normalised ratio**		
**MicroRNA**	**Accession no.**	**Fold change**	**Normal**	**Tumour**	***P*-value**
miR-874	MIMAT0004911	0.011	3.05E-04	3.36E-06	0.0463
miR-133a	MIMAT0000427	0.017	1.89E-02	3.14E-04	0.0033
miR-375	MIMAT0000728	0.035	3.95E-02	1.36E-03	0.0161
miR-204	MIMAT0000265	0.045	3.26E-02	1.47E-03	0.0055
miR-1	MIMAT0000416	0.054	1.88E-03	1.02E-04	0.024
miR-139-5p	MIMAT0000250	0.132	3.61E-02	4.75E-03	0.0044
miR-145	MIMAT0000437	0.167	6.57E-01	1.10E-01	0.0367
miR-143	MIMAT0000435	0.177	2.58E-01	4.57E-02	0.019
miR-486-3p	MIMAT0004762	0.183	2.26E-03	4.13E-04	0.0407
miR-146a	MIMAT0000449	0.253	1.09E+00	2.76E-01	0.0041
miR-410	MIMAT0002171	0.254	4.92E-04	1.25E-04	0.002
miR-126	MIMAT0000445	0.262	3.16E+00	8.28E-01	0.0037
miR-539	MIMAT0003163	0.278	4.00E-03	1.11E-03	0.005
miR-134	MIMAT0000447	0.308	1.69E-03	5.19E-04	0.0158
miR-218	MIMAT0000275	0.316	1.62E-02	5.13E-03	0.0329
miR-146b-5p	MIMAT0002809	0.337	5.18E-01	1.75E-01	0.0012
miR-140-3p	MIMAT0004597	0.341	2.04E-02	6.94E-03	0.0111
miR-30a-3p	MIMAT0000088	0.431	6.37E-02	2.75E-02	0.0089
miR-191	MIMAT0000440	0.489	6.22E-01	3.04E-01	0.0251
miR-186	MIMAT0000456	0.51	1.75E-01	8.90E-02	0.0309
miR-148a	MIMAT0000243	0.523	3.30E-02	1.73E-02	0.0334
miR-30e-3p	MIMAT0000693	0.525	4.95E-02	2.60E-02	0.0048
miR-29c	MIMAT0000681	0.552	7.94E-02	4.39E-02	0.0305

**Table 3:** The 18 downregurated genes in miR-874 transfectants

**Entrez gene ID**	**Gene name**	**Gene symbol**	**Log_2_ ratio**	**miR-874 target**
1373	Carbamoyl-phosphate synthetase 1, mitochondrial	*CPS1*	−1.85	2
3939	Lactate dehydrogenase A	*LDHA*	−1.75	—
1915	Eukaryotic translation elongation factor 1 *α* 1	*EEF1A1*	−1.45	—
5499	Protein phosphatase 1, catalytic subunit, *α* isozyme	*PPP1CA*	−1.25	1
5660	Prosaposin	*PSAP*	−1.23	—
80227	Proteasomal ATPase-associated factor 1	*PAAF1*	−1.22	1
567	*β*-2-microglobulin	*B2M*	−1.21	—
303	Annexin A2 pseudogene 1	*ANXA2P1*	−1.18	—
5223	Phosphoglycerate mutase 1 (brain)	*PGAM1*	−1.15	—
1303	Collagen, type XII, *α* 1	*COL12A1*	−1.09	1
3486	Insulin-like growth factor binding protein 3	*IGFBP3*	−1.08	—
2778	GNAS complex locus	*GNAS*	−1.08	—
55536	Cell division cycle associated 7-like	*CDCA7L*	−1.06	—
8667	Eukaryotic translation initiation factor 3, subunit H	*EIF3H*	−1.05	1
10916	Melanoma antigen family D, 2	*MAGED2*	−1.05	—
10618	*Trans*-Golgi network protein 2	*TGOLN2*	−1.03	2
4077	Neighbor of BRCA1 gene 1	*NBR1*	−1.02	1
343477	Heat shock protein 90 kD *β* (Grp94), member 3 (pseudogene)	*HSP90B3P*	−1.00	—
