# Supplementary material for: Tumour suppressive microRNA-874 regulates novel cancer networks in maxillary sinus squamous cell carcinoma
Source: Br J Cancer. 2011 Aug 16;105(6):833–41. doi: 10.1038/bjc.2011.311 (PMC3171017; doi:10.1038/bjc.2011.311)
Supplement: Supplementary Figure [file bjc2011311x1.ppt]

## Slide 1
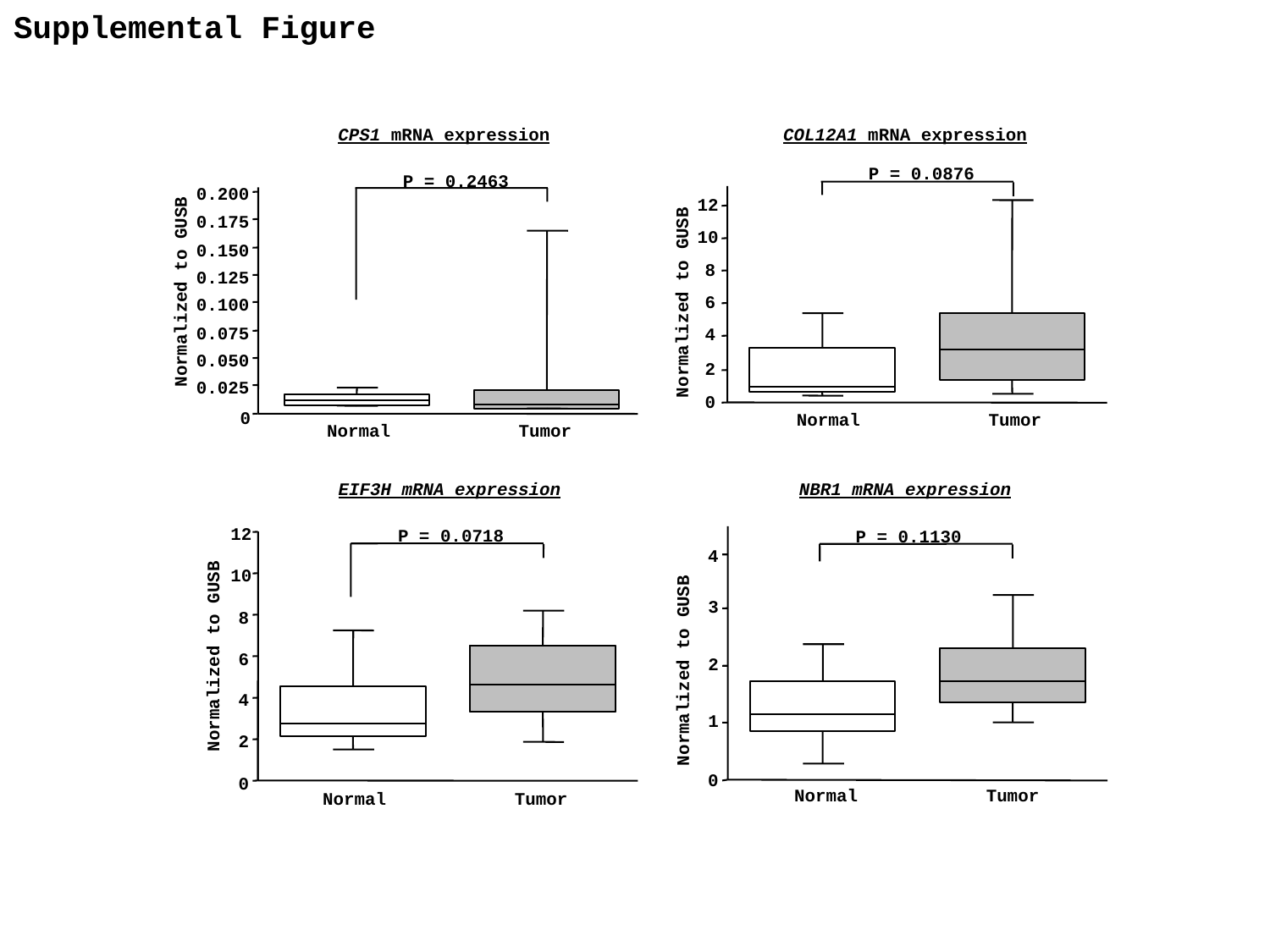

Supplemental Figure
CPS1 mRNA expression
COL12A1 mRNA expression
P = 0.0876
P = 0.2463
0.200
12
0.175
10
0.150
8
Normalized to GUSB
0.125
Normalized to GUSB
6
0.100
0.075
4
0.050
2
0.025
0
Normal
Tumor
0
Normal
Tumor
EIF3H mRNA expression
NBR1 mRNA expression
P = 0.0718
P = 0.1130
12
4
10
3
8
Normalized to GUSB
6
2
Normalized to GUSB
4
1
2
0
0
Normal
Tumor
Normal
Tumor
