# Supplementary material for: Tumour suppressive microRNA-874 regulates novel cancer networks in maxillary sinus squamous cell carcinoma
Source: Br J Cancer. 2011 Aug 16;105(6):833–41. doi: 10.1038/bjc.2011.311 (PMC3171017; doi:10.1038/bjc.2011.311)
Supplement: Supplementary Figure Legend [file bjc2011311x2.doc]

**FIGURE LEGENDS**

Supplementary Figure

Expression levels of four candidate genes of *miR-874* target were measured by real-time RT-PCR.

(A) *CPS1* mRNA expression levels in MSSCC clinical specimens. (B) *COL12A1* mRNA expression levels in MSSCC clinical specimens. (C) *EIF3H* mRNA expression levels in MSSCC clinical specimens. (D) *NBR1* mRNA expression levels in MSSCC clinical specimens. Real-time RT-PCR showed that there was no significant difference in the expression levels of the four genes between normal and tumour tissues. *GUSB* was used as an internal control.
