# Supplementary material for: Tumour suppressive microRNA-874 regulates novel cancer networks in maxillary sinus squamous cell carcinoma
Source: Br J Cancer. 2011 Aug 16;105(6):833–41. doi: 10.1038/bjc.2011.311 (PMC3171017; doi:10.1038/bjc.2011.311)
Supplement: Supplementary Document [file bjc2011311x3.doc]

**Supplemental document**

***PPP1CA* 3’UTR, 353bp (wild type)**

ccccgcacaccaccctgtgccccagatgatggattgattgtacagaaatcatgctgccatgctgggggggggtcaccccgacccctcaggcccacctgtcacggggaacatggagccttggtgtatttttcttttctttttttaatgaatcaatagcagcgtccagtcccccagggctgcttcctgcctgcacctgcggtgactgtgagcaggatcctggggccgaggctgcagctcagggcaacggcaggccaggtcgtgggtctccagccgtgcttggcctcagggctggcagccggatcctggggcaacccatctggtctcttgaataaaggtcaaagctggattctcgc

***PPP1CA* 3’UTR, 346bp (Position 237-243 deletion)**

Ccccgcacaccaccctgtgccccagatgatggattgattgtacagaaatcatgctgccatgctgggggggggtcaccccgacccctcaggcccacctgtcacggggaacatggagccttggtgtatttttcttttctttttttaatgaatcaatagcagcgtccagtcccccagggctgcttcctgcctgcacctgcggtgactgtgagcaggatcctggggccgaggctgcagct-------acggcaggccaggtcgtgggtctccagccgtgcttggcctcagggctggcagccggatcctggggcaacccatctggtctcttgaataaaggtcaaagctggattctcgc
